# New COVID-19 Transmission after the First Vaccine Dose at Skilled Nursing Facilities in Nebraska

**DOI:** 10.1017/ash.2021.36

**Published:** 2021-07-29

**Authors:** Ishrat Kamal-Ahmed, FNU Kanishka, Derry Stover, Matthew Donahue, Yi Du, Derek Julian, Dan German

## Abstract

Group Name: DHHS Epi

**Background:** The inoculation with SARS-CoV-2 vaccine at long-term care facilities (LTCFs) in Nebraska began on December 28, 2020, as part of the Centers for Disease Control and Prevention (CDC) Pharmacy Partnership for Long-Term Care Program.^1^ As of February 5, 2021, 159 skilled nursing facilities (SNFs) had completed their first vaccine clinic, and 7,271 residents and 6,768 staff had received the first dose of the 2-dose series. Surveillance data before vaccination (December 21–27, 2020) and after the first vaccination dose (January 25–31, 2021) indicate that the weekly SARS-CoV-2 positivity rate at SNFs decreased from 1.18% to 0.42% for residents and 0.54% to 0.11% for staff.^2,3,4^ In this study, we examined the perceived decrease in new transmission initiated by the first dose of vaccine at SNFs. **Methods:** We analyzed the data with separate logistic regressions for residents and staff. We included 145 SNFs that completed their first vaccine clinic, and we used the Federal and Pharmacy Partnership database for the number of residents and staff that received the first dose of vaccine at the first vaccine clinic. We followed the SNFs for 21 days after the first vaccine clinic from December 28, 2020, through February 5, 2021, for any first-time SARS-CoV-2–positive cases. The National Healthcare Safety Network (NHSN) database was used to collect the information on the number of residents present at the facility on the day of the first vaccine clinic, if available, or days before in the same week as the first vaccine clinic. The staff count for each facility was extracted from Nebraska Licensure for LTCFs. We collected new case information from the state surveillance, the NHSN, and the Test-Nebraska platform. **Results:** The mean resident vaccine coverage was 80% and the median staff vaccine coverage was 43%. We found a reverse association between staff vaccine coverage and new positive staff cases. For each percentage increase in staff vaccine coverage, the odds of having a new staff positive case 7 days and 14 days after the first vaccine clinic decrease by 26% and 48%, respectively. No association between coverage and new resident transmission was detected. Possible confounding exists when infected residents might have tested positive 7–14 days after the first vaccine clinic who were not affected by the vaccine. **Conclusions:** Although we observed the association between lower case count with increased facility-level vaccine coverage, we would need to wait for the administration of the second dose of vaccine before assessing the level of association between coverage and new transmission. Further initiatives are warranted to increase the suboptimal vaccine coverage for staff.

**Funding:** No

**Disclosures:** None

